# Supplementary material for: Assessing the Antimicrobial Properties of Honey Protein Components through In Silico Comparative Peptide Composition and Distribution Analysis
Source: Antibiotics (Basel). 2023 Apr 28;12(5):830. doi: 10.3390/antibiotics12050830 (PMC10215533; doi:10.3390/antibiotics12050830)
Supplement: Supplementary file 1 [file antibiotics-12-00830-s001.zip › S1-2_Supplementary data.pdf]

S1. Alignment results

- Data reported in the spreadsheet were generated with the following command:

```
diamond blastp --db reference_database.dmnd --query querry_file.fasta --
out reference_querry_diamond.tsv --outfmt 6 qseqid sseqid pident length
mismatch gapopen qstart qend sstart send evalue bitscore qlen qseq stitle
slen sseq staxids sscinames sskingdoms --threads 6 --log
```

- Spreadsheet tabs are named according to the following format:

*reference_querry_*diamond

- Spreadsheet column are named as fallow:

```
qseqid sseqid pident length mismatch gapopen qstart qend sstart send
evalue bitscore qlen qseq stitle slen sseq staxids sscinames sskingdoms
```

Detailed description is available at the software distribution site:

https://github.com/bbuchfink/diamond
https://github.com/bbuchfink/diamond/wiki/3.-Command-line-options

S2. Additional plots.

- Figure S2.1. Alignment of the AMPs in the APD3 database: hits distribution against APD3 database, size of the point is proportional to the hit count.
- Figure S2.2. Alignment of the DBAASP against APD3 database: size of the point is proportional to the hit count.
- Figure S2.3. Alignment of the AMPs in the DBAASP database: hits distribution against DBAASP database, size of the point is proportional to the hit count.
- Figure S2.4. Histogram of the AMPs sequences percent identity: content of the DBAASP database compared against itself.
