# Supplementary material for: Assessing the Antimicrobial Properties of Honey Protein Components through In Silico Comparative Peptide Composition and Distribution Analysis
Source: Antibiotics (Basel). 2023 Apr 28;12(5):830. doi: 10.3390/antibiotics12050830 (PMC10215533; doi:10.3390/antibiotics12050830)
Supplement: Supplementary file 1 [file antibiotics-12-00830-s001.zip › S2_Additional plots.pdf]

S2. Additional plots.

- Figure S2.1. Alignment of the AMPs in the APD3 database: hits distribution against APD3 database, size of the point is proportional to the hit count.
- Figure S2.2. Alignment of the DBAASP against APD3 database: size of the point is proportional to the hit count.
- Figure S2.3. Alignment of the AMPs in the DBAASP database: hits distribution against DBAASP database, size of the point is proportional to the hit count.
- Figure S2.4. Histogram of the AMPs sequences percent identity: content of the DBAASP database compared against itself.

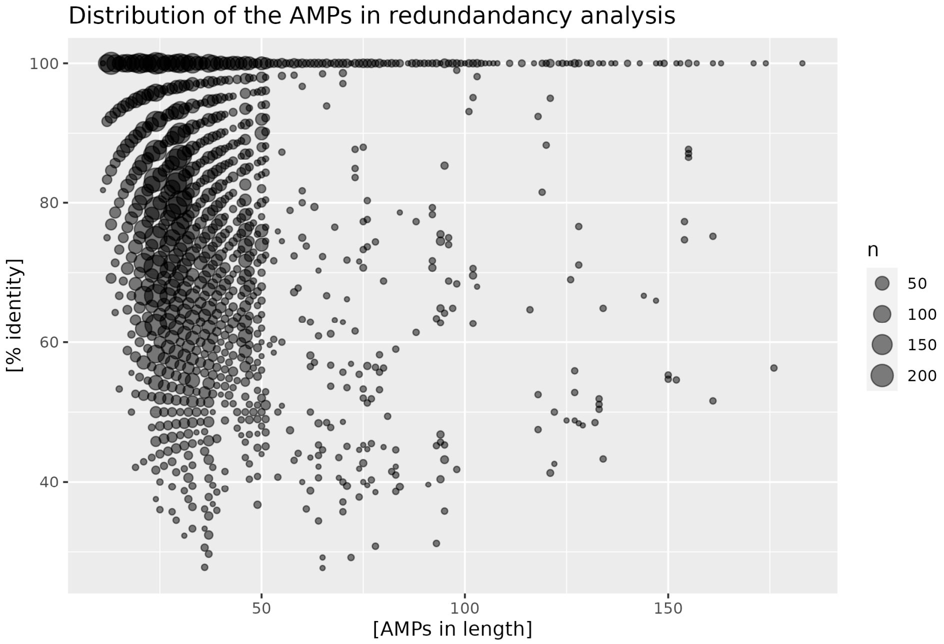

Figure S2.1. Alignment of the AMPs in the APD3 database: hits distribution against APD3 database, size of the point is proportional to the hit count.

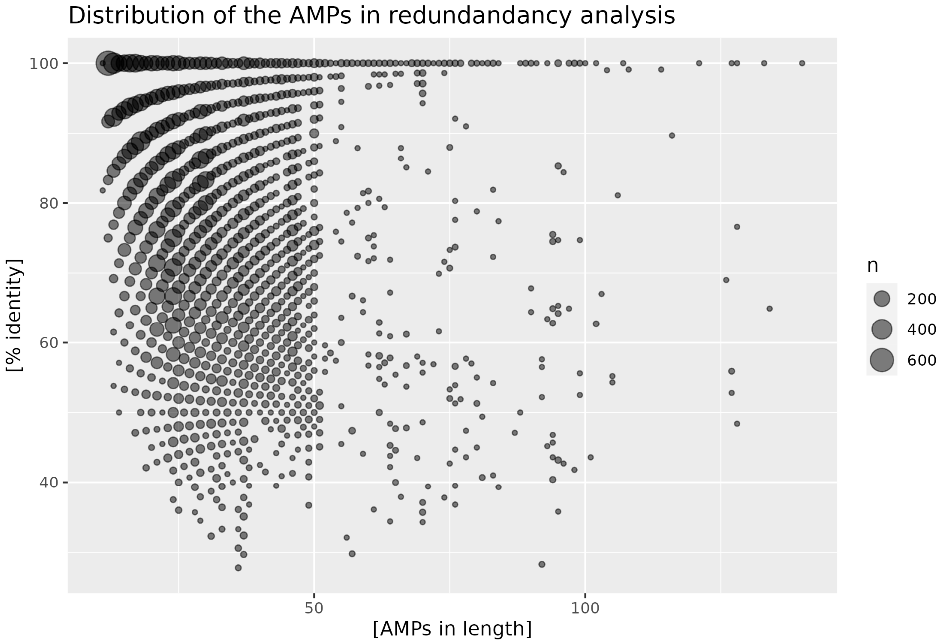

Figure S2.2. Alignment of the DBAASP against APD3 database: size of the point is proportional to the hit count.

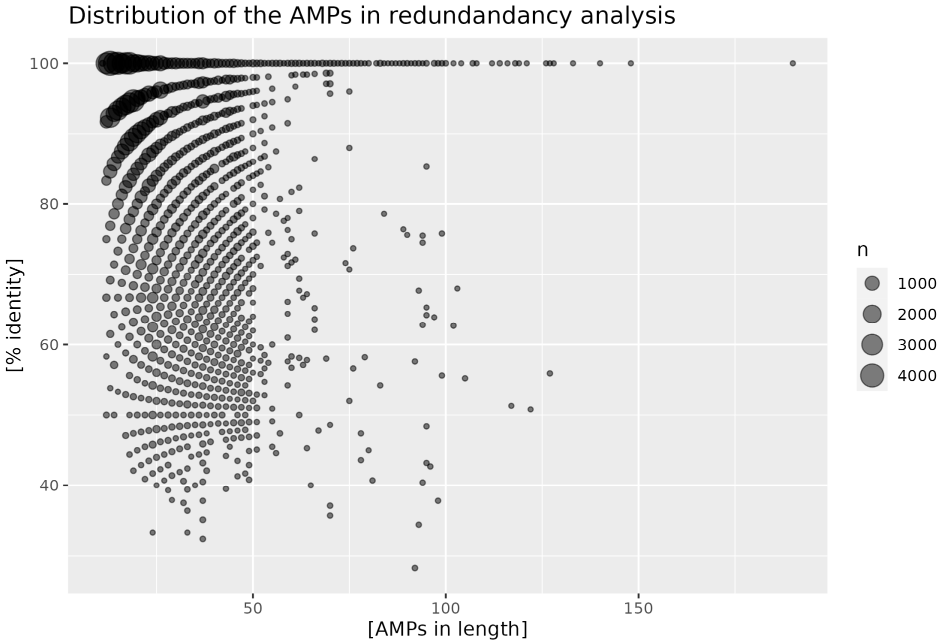

Figure S2.3. Alignment of the AMPs in the DBAASP database: hits distribution against DBAASP database, size of the point is proportional to the hit count.

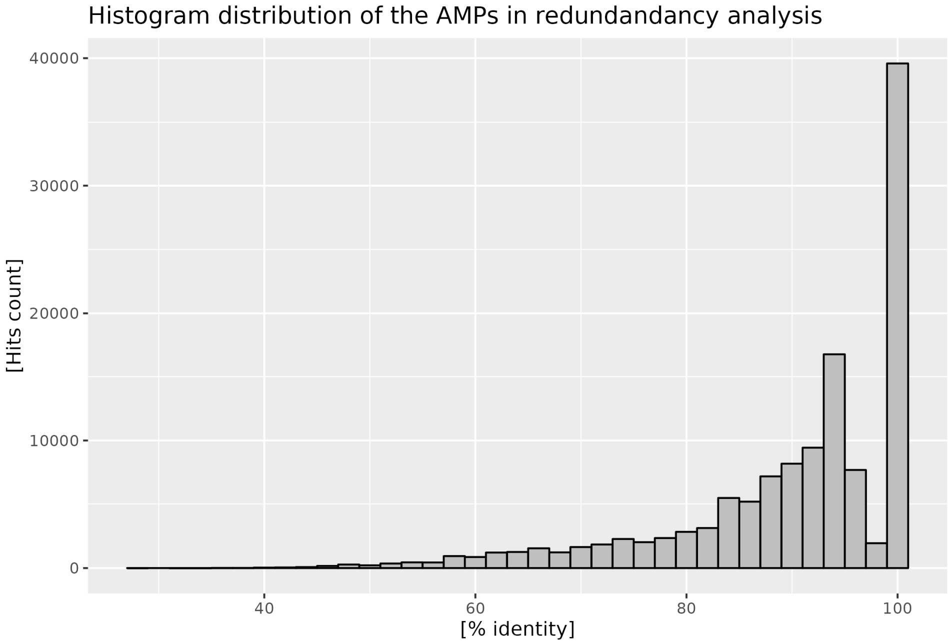

Figure S2.4. Histogram of the AMPs sequences percent identity: content of the DBAASP database compared against itself.
